# Urea Gel Electrophoresis in Studies of Conformational Changes of Transferrin on Binding and Transport of Non-Ferric Metal Ions

**DOI:** 10.3390/gels8010019

**Published:** 2021-12-27

**Authors:** Aviva Levina, Boer Wang, Peter A. Lay

**Affiliations:** 1 School of Chemistry, University of Sydney, Sydney, NSW 2006, Australia; bwan8562@uni.sydney.edu.au; 2 Sydney Analytical, University of Sydney, Sydney, NSW 2006, Australia

**Keywords:** transferrin, urea-PAGE, iron, chromium, vanadium, indium, gallium, titanium

## Abstract

Transferrin (Tf) is a crucial transporter protein for Fe(III), but its biological role in binding other metal ions and their delivery into cells remain highly controversial. The first systematic exploration of the effect of non-Fe(III) metal ion binding on Tf conformation has been performed by urea-polyacrylamide gel electrophoresis (urea-PAGE), which is commonly used for nucleic acids but rarely for proteins. Closed Tf conformation, similar to that caused by Fe(III)-Tf binding, was formed for In(III), V(III) or Cr(III) binding to Tf. In all these cases, metal distribution between Tf lobes and/or the rate of metal release under acidic conditions differed from that of Fe(III)-Tf. By contrast, Ga(III) and V(IV) did not form closed Tf conformation under urea-PAGE conditions. Apart from Fe(III), only In(III) was able to increase the proportion of closed Tf conformation in whole serum. These results suggest that Tf is unlikely to act as a natural carrier of any metal ion, except Fe(III), into cells but can reduce toxicity of exogenous metal ions by binding them in serum and preventing their entry into cells.

## 1. Introduction

The transferrin (Tf) family of proteins, which evolved for the purpose of safe and efficient transport of Fe(III) into the cells of most animals, is widely studied as classical examples of high affinity metal-protein binding and cellular uptake via receptor-mediated endocytosis [1,2]. Human Tf is a ~80 kDa glycoprotein with two structurally similar lobes (*C*-lobes and *N*-lobes) that bind Fe(III) in a chemically identical manner (2 × Tyr + Asp + His + synergistic CO_3_^2−^) [1,2]. Binding of Fe(III) to both Tf lobes results in a closed Tf conformation that binds tightly to Tf receptors, such as TfR1, on the cell membrane and delivers Fe into the cell via an elaborate endocytotic mechanism (Tf cycle) [1,2,3]. There are subtle but biologically important differences between the two Tf lobes in the thermodynamics and kinetics of Fe(III) binding and release [4]. The ability of Tf to bind many other (particularly trivalent) metal ions at the Fe(III) binding site [5] and the fact that human blood Tf is normally only ~30% Fe(III) saturated [6] resulted in the hypothesis that Tf can act as a carrier of other biological and non-biological metal ions into the cells [5,7,8]. However, growing evidence suggests that, in most cases, binding of metals other than Fe(III) to Tf results in changes in Tf conformation that disrupt the Tf cycle and prevent their cellular uptake so that Tf is more likely to protect cells from toxicity of these metal ions [9,10,11]. Essentiality of some transition metal ions, particularly V and Cr, for human nutrition and the role of Tf in their metabolism remain as topics of hot debate [9,10,12,13]. 

**Figure 1:**
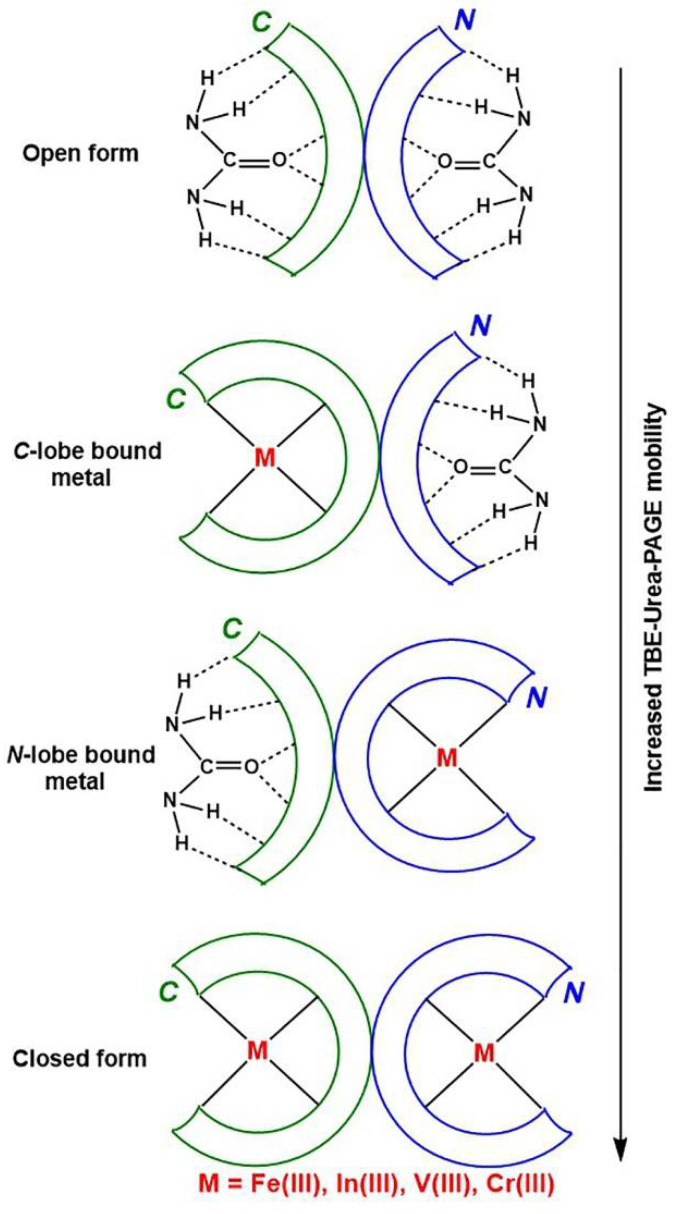
A schematic representation of the role of urea in changing electrophoretic mobility of various Tf conformations (based on data of [4,16]). Designations: *C* and *N* are the two lobes of Tf, TBE is a Tris-borate-EDTA buffer (where Tris is tris(hydroxymethyl)aminomethane, EDTA = *N*,*N*,*N*′*N*′-ethylenediaminetetraacetic acid) and PAGE is polyacrylamide gel electrophoresis.

In the field of separation of biological macromolecules by polyacrylamide gel electrophoresis (PAGE), sodium dodecyl sulfate (SDS) and urea have long been considered the best denaturing agents for proteins and nucleic acids, respectively [14]. A serendipitous observation was made 45 years ago that typical separation conditions of nucleic acids (6 M urea, pH 8.3 buffer) could be used to distinguish the Fe(III) binding status of two lobes of Tf [15], and the method has since been widely used [4]. The ability of urea to form hydrogen bonds with the peptide backbone of proteins [16] results in its preferential binding to the open (metal-free) lobes of Tf, which slows its movement through the gel (Figure 1) [4]. Since the *C*-lobe is more compact in shape than the *N*-lobe (the two lobes contain eleven and eight disulfide bonds, respectively) [4], the partially open conformation with the *C*-lobe open moves through the gel faster than that with the *N*-lobe open (Figure 1).

Although urea-PAGE has been used occasionally to study the binding of non-Fe metal ions to Tf [10,17,18,19,20], the scope of this technique for elucidation of the roles of Tf in ‘alternative’ metal metabolism [8,11] has not been explored systematically. This communication demonstrates the ability of this technique to test how various metal ions binding to Tf in a Fe(III)-like manner change the conformation of metalloprotein. This enables the prediction of the capability of resultant metal-Tf complexes to bind to TfR1 and to enter the cell [3,9,10]. The resultant information enables some of the long-standing controversies to be addressed regarding the biological roles of transition metal ions [21,22]. 

## 2. Results and Discussion

### 2.1. Conformation of Cr(III)-Tf Complexes Depends on the Conditions of Their Formation

**Figure 2:**
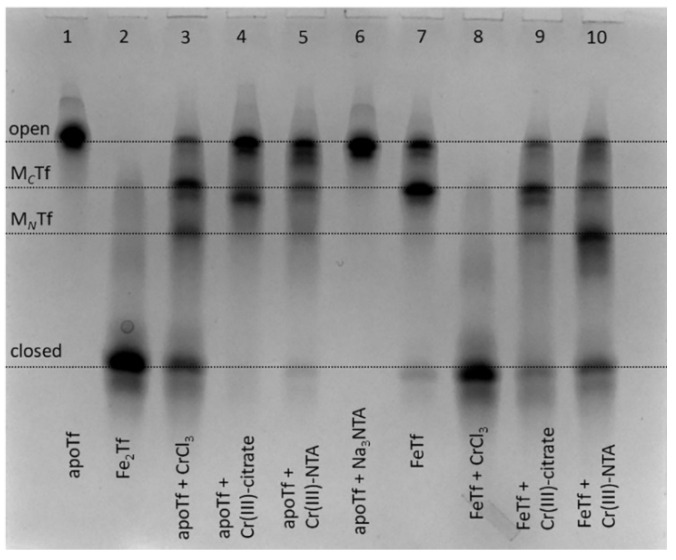
Urea-PAGE of reaction mixtures containing 30 μM apoTf or FeTf and 150 μM of Cr(III) complexes (aquated CrCl_3_, Cr(III)-citrate or Cr(III)-NTA, see Section 4 for details) in the binding buffer (20 mM HEPES, 25 mM NaHCO_3_, 140 mM NaCl, pH 7.4) [3] after 24 h of reaction at 310 K. Lanes 1, 2, 6 and 7 contain control samples without Cr(III). All samples were diluted to ~5 μM Tf with the binding buffer before loading into the gel. Four main Tf conformations (Figure 1) are marked (M*_C_*Tf and M*_N_*Tf contain one metal ion bound to *C*-lobes or *N*-lobes, respectively).

Binding of Cr(III) to Tf is achieved by the reactions of apoTf (metal-free Tf) with 2–10 molar excess of Cr(III) (usually aquated CrCl_3_) in a neutral aqueous buffer that contains 25 mM HCO_3_^−^ (a synergistic anion required for metal binding to Tf) [9,23,24], but the reaction outcome can be affected by hydrolysis of Cr(III) salt [23]. We report the first use of urea-PAGE as a convenient method to test various conditions for the generation of Cr(III)-Tf complexes; a typical example is shown in Figure 2.

In addition to using CrCl_3_ as a Cr(III) source [9,23,24], complexation of Cr(III) with citrate or nitrilotriacetic acid (NTA) was used to prevent Cr(III) hydrolysis in neutral aqueous media by analogy with classical techniques of Tf loading with Fe(III) and other metal ions [25,26]. These complexants also bind to non-Fe(III) metals in Fe(III)-binding sites of Tf, as shown in the crystal structures of Bi^III^(NTA)FeTf [27] and Ti^IV^(citrate)Tf [26]. Of all the conditions shown in Figure 2, only the reaction of monoferric Tf (FeTf) with 5-fold molar excess of CrCl_3_ resulted in the formation of fully closed Tf conformation (lane 8). Although binding of a non-Fe metal ion to Tf that has one of the lobes ocuppied with Fe is a realistic situation in vivo [10,27,28], this is unlikely to occur for Cr(III) in blood serum due to its competing binding to albumin and small bioligands, such as citrate [21]. All other conditions shown in Figure 2 resulted in the formation of various mixtures of Tf conformations, with only small proportions of closed Tf conformation, which is consistent with the previous finding [9] that binding to Tf inhibits Cr(III) uptake by cells. 

Notably, while FeTf shows predominant metal binding to the *C*-lobe (lane 7), Cr(III) tended to bind to the *N*-lobe under some conditions (lanes 3 and 10). By contrast, a recently reported crystal structure [24] showed Cr(III) binding to the *C*-lobe of Tf, with the *N*-lobe remaining unoccupied, although this used a non-physiological synergistic anion, malonate. Further studies with the use of recombinant Tf forms that have only one lobe available for metal binding [4] will be required in order to clarify the preference of Cr(III) binding to *C*-lobe vs. *N*-lobe of Tf under various conditions. The results in Figure 2, together with the literature data [9,23], show that the binding process of Cr(III) to Tf is more complicated than that of Fe(III) due to the kinetic inertness of Cr(III), and conformation of the formed Cr(III)-Tf adducts can depend on a number of factors, including reaction time and the nature of synergistic anions. This is an indication that Tf is unlikely to be the protein that naturally evolved for Cr(III) transport, as suggested previously [8], although it can play a role in the binding and detoxification of exogenous Cr(III) in blood [9,23]. Moreover, the high level of Tf produced during immune system response to bacterial and fungal infections [1,2] may also have a role in detoxifying the high levels of Cr accumulated by many of these pathogens [29,30].

### 2.2. Binding of In(III), but Not Ga(III)—Results in Closed Tf Conformation

**Figure 3:**
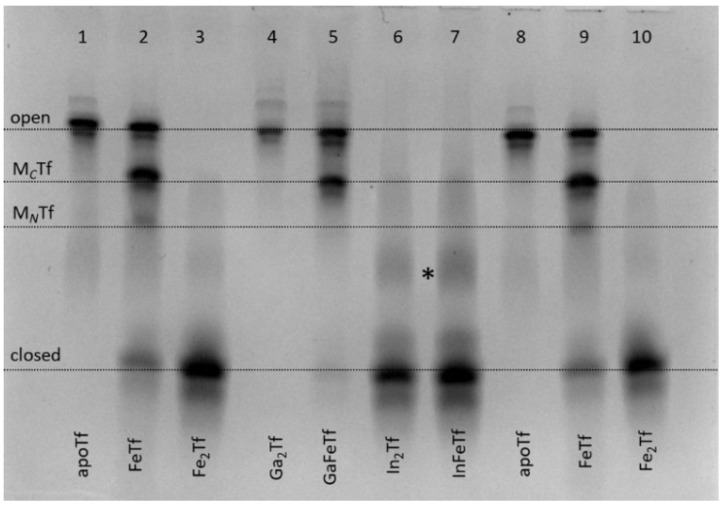
Urea-PAGE of purified Ga(III)-Tf (lanes 4 and 5) and In(III)-Tf (lanes 6 and 7) in comparison with control samples (apoTf, lanes 1 and 8; FeTf, lanes 2 and 9; and Fe_2_Tf, lanes 3 and 10). See Section 4 for sample preparation. All samples were diluted to ~5 μM Tf with the binding buffer (20 mM HEPES, 25 mM NaHCO_3_, 140 mM NaCl, pH 7.4) [3] before loading into the gel. Four main Tf conformations (Figure 1) are marked (M*_C_*Tf and M*_N_*Tf contain one metal ion bound to *C*-lobes or *N*-lobes, respectively). A possible additional band for In(III)-Tf is marked with an asterisk.

For several decades, development of Ga(III) complexes as anticancer drugs was based on the assumption that Ga(III), due to its close chemical similarity to Fe(III), can efficiently bind to Tf and enter cells through the Tf cycle, which results in Fe starvation of rapidly growing cancer cells [31]. Medicinal use of In(III), the closest chemical analogue of Ga(III), has also been proposed based on the same assumption, but it remains relatively unexplored [32]. Both metal ions (^67^Ga, ^68^Ga and ^111^In isotopes) are widely used in medical imaging, which is also thought to rely at least in part on their binding to Tf [31,32]. Unexpectedly, Ga(III) binding to apoTf or FeTf did not result in closed Tf conformation that would be stable under urea-PAGE conditions, while In(III) readily formed such conformation under a variety of conditions, including the use of InCl_3_, In(III)-citrate or In(III)-NTA as metal sources; typical examples are shown in Figure 3. 

**Figure 4:**
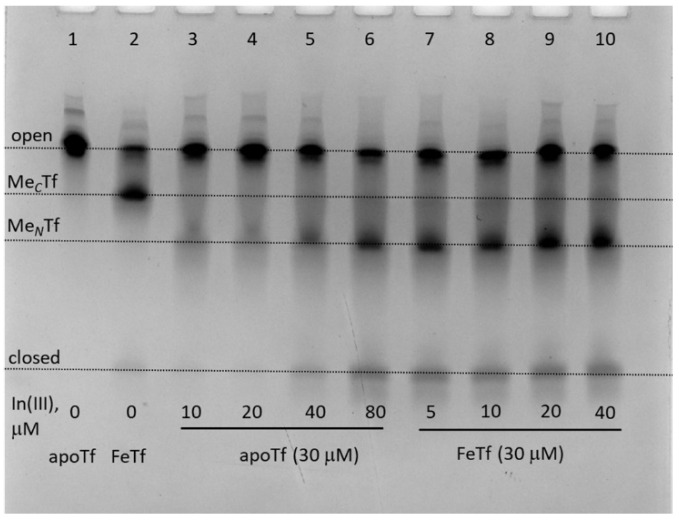
Urea-PAGE of the mixtures containing 30 μM of apoTf or FeTf and varied concentrations of In(III)-NTA (see Section 4 for details) in the binding buffer (20 mM HEPES, 25 mM NaHCO_3_, 140 mM NaCl, pH 7.4) [3] reacted for 24 h at 310 K. All samples were diluted to ~5 μM Tf with the before loading into the gel. Four main Tf conformations (Figure 1) are marked (M*_C_*Tf and M*_N_*Tf contain one metal ion bound to *C*-lobes or *N*-lobes, respectively).

The greater stability of In(III)-Tf binding, compared with Ga(III)-Tf, is consistent with recent mass spectrometry data [33,34], although the stability of Ga(III)-Tf adducts can also decrease due to the formation of stable [Ga(OH)_4_]^−^ anions under urea-PAGE conditions (pH 8.3) [35]. Close inspection of Figure 3 reveals some differences in Tf conformation in the presence of Ga(III) or In(III) compared with their Fe(III) analogues. For instance, a sample of GaFeTf (lane 5) showed the absence of a significant M*_N_*Tf band, in contrast to FeTf samples (lanes 2 and 9), while there was a likely additional partially open conformation for In(III)-Tf (lanes 6 and 7, marked with as asterisk). A difference in metal distribution between In(III)-Tf and Fe(III)-Tf complexes was further highlighted in the reactions of varied In(III)-NTA concentrations with apoTf or FeTf, which showed preferential In(III) binding to the *N*-lobe of the protein, in contrast with the predominantly *C*-lobe binding of Fe(III)-Tf (Figure 4).

It is likely that for In(III), similarly for Cr(III), the Tf binding pattern is more complicated than that of Fe(III) due to the relative kinetic inertness of the metal ion [9,23]. This complication should be kept in mind when medicinal applications of In(III), related to its Tf binding, are considered [32,33]. On the other hand, the likely low stability of Ga(III)-Tf under biologically relevant conditions (Figure 3) [33,34] means that alternative mechanisms of Ga(III) biological activity, such as the interference with Ca(II) and carboxylic acid metabolism [36,37], can be more important than Ga(III)-Tf binding.

### 2.3. Strong V(III)-Tf and V(IV)-Tf Bindings Are Retained under Endosomal Conditions

**Figure 5:**
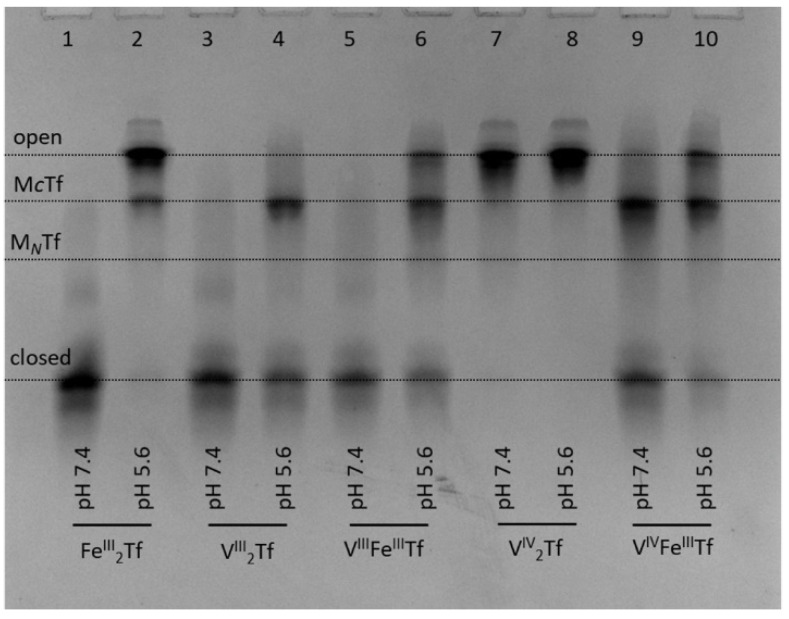
Urea-PAGE of the purified samples of V^III^_2_Tf, V^III^FeTf, V^IV^_2_Tf and V^IV^FeTf (see Section 4 for details) that reacted under the conditions mimicking the extracellular (20 mM HEPES, 25 mM NaHCO_3_, 140 mM NaCl, pH 7.4) and endosomal (100 mM MES, 300 mM KCl, 0.10 mM citrate, 1.0 mM ascorbate, pH 5.6) stages of the Tf cycle [3] for 5 min at 295 K. Initial Tf concentration in the reaction mixtures was 30 μM, and samples were diluted 6-fold with corresponding buffers before loading into the gel. Four main Tf conformations (Figure 1) are marked (M*_C_*Tf and M*_N_*Tf contain one metal ion bound to *C*-lobes or *N*-lobes, respectively).

The ability of all three biologically relevant oxidation states of V (V(III), V(IV) and V(V)) to bind to Tf is well established [13,38]. Recently, it was demonstrated that V(IV)-Tf and V(V)-Tf bindings result in a drastic decrease in cellular uptake and biological activity of V complexes [10,39]. Most intriguingly, Tf binding stabilizes V(III), which is air-sensitive under ambient conditions but is likely to form under reducing conditions of whole blood [19,20,40]. Strong V(III)-Tf binding is facilitated by the close chemical similarity between V(III) and Fe(III) [13,38]. Our urea-PAGE experiments confirmed previous reports [20] that V(III)-Tf adducts assume closed conformation, similar to that of Fe(III)-Tf, at pH 7.4 (lanes 3 and 5 in Figure 5).

In addition, we performed the first urea-PAGE study of the stability of V(III)-Tf complexes under endosomal conditions (i.e., those resulting in the release of Fe from Tf inside the cell) [1,2,3], similar to that reported previously for Fe(III)-Tf complexes [4]. Note that endosomal conditions used before [4] were modified to include citrate (0.10 mM) and ascorbate (1.0 mM) that are likely to act as a complexant and a reductant of Fe(III), respectively, in the cellular Tf cycle [3,34]. As shown in Figure 5, our endosomal conditions resulted in efficient opening of the closed Fe_2_Tf form, indicating Fe release (lanes 1 and 2) [4]. By contrast, only partial opening occurred under the same conditions for V^III^_2_Tf (lanes 3 and 4) or V^III^FeTf (lanes 5 and 6). In agreement with previous data [10,20], V^IV^_2_Tf did not form a closed conformation; thus, there were no significant differences in its urea-PAGE patterns at pH 7.4 or pH 5.6 (lanes 7 and 8). A mixed V^IV^FeTf complex showed a higher proportion of closed Tf conformation at pH 7.4 compared with FeTf (lane 9 in Figure 5 vs. lane 2 in Figure 4), and the opening of Tf conformation under endosomal conditions was retarded compared with that of Fe_2_Tf (lanes 10 vs. lane 2 in Figure 5). Similar results were obtained using different preparations of V^III^_2_Tf and V^III^FeTf that contained mixtures of fully closed and partially opened Tf conformations at pH 7.4 (Figure S1 in Supplementary Material).

These results suggest that mixed V^III^FeTf and V^IV^FeTf adducts can play a role in V delivery into mammalian cells [10,13,19,20,41]. However, their reduced reactivity under endosomal conditions (Figure 5) means that Tf-bound V species can return to extracellular space via the Tf cycle rather than remaining in the cell [10], which results in an inhibitory effect of Tf on cellular V uptake [10,39]. 

For the V(III)-Tf species, the reduced efficiency of release of V under endosomal conditions is likely to be due to the difficulty of reducing V(III) to V(II) under endosomal conditions that cause Fe(III) reduction to Fe(II) [1,2,3]. In addition, any d^3^ V(II)-Tf is likely to be much more kinetically inert than high-spin d^5^ Fe(II)-Tf, hence hindering metal release. A lower efficiency of metal release under endosomal conditions, compared with Fe(III)-Tf, has also been reported for Cr(III)-Tf [9,34], In(III)-Tf [34] and Ti(IV)-Tf [34], although biological significance of these findings for Cr(III)-Tf has been disputed [42]. The question about essentiality of V to mammals (as opposed to some bacteria, algae or marine invertebrates that are known to require V for life) remains open [13]. The present results suggest that V^III^_2_Tf and V^III^FeTf could play a role in V metabolism, but the biochemistry of these V(III) species needs to be investigated in much more detail, including the use of recombinant Tf forms that have only one lobe available for metal binding [4]. 

### 2.4. Urea-PAGE Can Be Used to Test Metal-Tf Binding in Blood Serum

**Figure 6:**
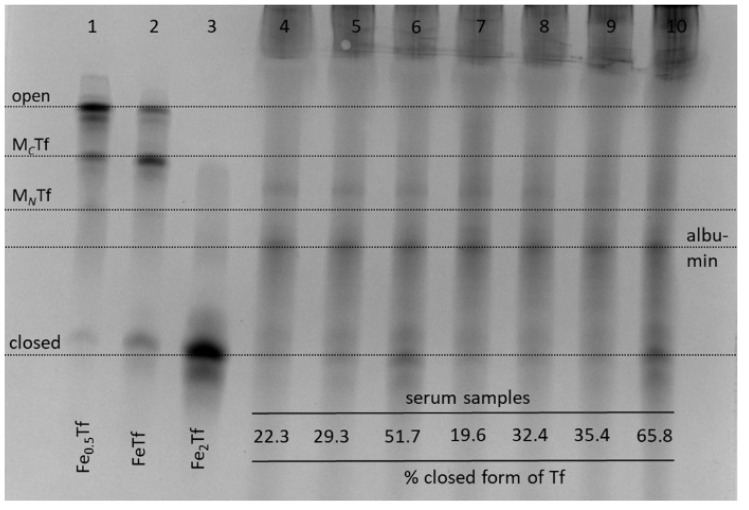
Typical urea-PAGE results for albumin-depleted human serum samples in the presence or absence of metal complexes: lane 4, no metals added; lane 5, Fe(III)-citrate; lane 6, Fe(III)-NTA; lane 7, Cp_2_TiCl_2_; lane 8, VOSO_4_·5H_2_O; lane 9, Na_3_VO_4_; lane 10, In(III)-citrate. Whole serum was incubated with 50 μM of metal complexes for 4 h at 310 K, followed by the removal of excess albumin with Affi-Gel Blue columns (see Section 4). Lanes 1–3 show control Tf samples with various Fe saturation in an aqueous buffer (20 mM HEPES, 25 mM NaHCO_3_, 140 mM NaCl, pH 7.4) [3]. Four main Tf conformations (Figure 1) are marked based on control samples (M*_C_*Tf and M*_N_*Tf contain one metal ion bound to *C*-lobes or *N*-lobes, respectively) and assignment of the albumin band is based on published data [10]. The relative content of closed Tf form in serum samples (shown at the bottom of the gel) was determined by image processing with BioRad ImageLab 5.2 software (Figure S2 in Supplementary Materials).

For most non-Fe metal ions and medicinal metal complexes, there is a strong competition for metal binding in blood serum between Tf and other serum proteins, particularly albumin [10,11,13,21,43]. In our preliminary experiments, undiluted human serum was reacted with 50 μM of metal complexes, which is within the range of concentrations observed for anticancer metal complexes in clinical use [44], for 4 h at 310 K. This was followed by the removal of most albumin using affinity columns (see Section 4) and loading the samples into urea gels (Figure 6; see Figure S2 in Supplementary Material for data processing).

There was an expected increase in relative abundance of closed Tf form in the presence of added Fe(III)-NTA (lane 6, Figure 6), which is known to load Fe(III) to Tf with high efficiency [25]. Among the other metal complexes tested (Figure 6), only In(III)-citrate caused a visible increase in the content of closed Tf conformation (lane 10). While not reported here, the binding of Ru(III) anticancer drugs to Tf also results in the disruption of Tf cycle, which has been attributed to differences in Tf conformation compared to Fe_2_Tf [45]. Another noteworthy observation was the lack of serum Tf binding for titanocene dichloride (Cp_2_TiCl_2_), which is a widely studied anticancer metal complex that is thought to rely on Tf for delivery into cells [46]. However, Cp_2_TiCl_2_ is also known to bind extensively to serum albumin [43]. Taken together, Figure 6 shows that the use of urea-PAGE to the study of metal-Tf binding is not limited to pure protein samples and can be applied to whole serum, as well as to cell culture medium [10].

### 2.5. Scope and Limitations of Urea-PAGE and Other Techniques for the Studies of Metal-Tf Binding

As shown in previous sections, urea-PAGE provides a simple and inexpensive technique for assessing the ability of metal ions and metal-based drugs to bind to Tf with the formation of a stable closed conformation and to release metal ions under conditions that mimic cellular endosomes. These data provide key information for predicting if metal ion can be delivered into cells by Tf [9,10,27]. An important limitation of this technique is the partial change of protein conformation during gel electrophoresis (pH 8.3), which can result in re-closure of open Tf conformations that were formed under endosome-mimicking conditions [4]. In addition, some metal-Tf complexes, such as Ti(IV)-Tf, are likely to enter cells even without the formation of closed Tf conformation [26]. It is, therefore, important to use this method in combination with other techniques, each of which has its own advantages and limitations. For instance, Cr(III) and V(IV) binding to Tf were studied mostly by electronic absorption (UV-vis), circular dichroism (CD) and electron paramagnetic resonance (EPR) spectroscopies [13,19,20,23,38,42,47]. These techniques typically use very high protein concentrations (≥0.5 mM), which is expensive and non-physiological, while urea-PAGE experiments required low volumes (50–100 μL) of Tf solutions at a physiological concentration (30 μM) [6]. Studies of Ga(III) and In(III) binding to Tf were performed by mass spectrometry [33,34] that relies on detection of metal-Tf complexes in the gas phase, which is far removed from biological conditions. Important additional techniques include the assessment of protein conformation by small angle X-ray scattering (SAXS) analysis [47] and the studies of Tf binding to TfR1 using biolayer interferometry (BLI) [3,9,10,45], or surface plasmon resonance (SPR) [43] techniques. The effect of metal-Tf binding on cytotoxicity and cellular metal uptake has been measured by using cell culture techniques [9,10,26,39]. Finally, the gels such as those shown in Figure 2, Figure 3, Figure 4, Figure 5 and Figure 6 can be blotted onto polymer membranes and used for metal imaging by X-ray fluorescence mapping (XFM), although the accessibility of this technique is limited by the need to use synchrotron radiation [48].

## 3. Conclusions

Urea-PAGE is a classical technique used for the separation of nucleic acids, but its use for separation of different conformations of the same protein (not achievable by the common protein separation technique, SDS-PAGE) is relatively unknown. Here, we provide the first systematic exploration of the use of urea-PAGE for studies on non-native metal ions binding to the crucial Fe(III) transport protein, transferrin (Tf). It was demonstrated that Cr(III), In(III) or V(III) binding to Tf can, under certain condition, result in closed protein conformation, similar to that formed by Fe(III)-Tf, while no closed conformation was observed for Ga(III) or V(IV). However, binding of Cr(III), In(III) or V(III) to Tf resulted in a different metal distribution between protein lobes and/or slower rates of metal release from Tf under endosome-mimicking conditions, compared with Fe(III)-Tf. Non-Fe(III) metal ions, except for In(III), are also unlikely to affect the distribution of Tf conformations in whole serum. These results suggest that Tf is unlikely to act as a natural carrier of any metal ion, except Fe(III) and perhaps to a lesser extent V(III), into cells but can reduce toxicity of exogenous metal ions by binding them in serum and preventing their entry into cells. As a simple and inexpensive technique, urea-PAGE is a useful addition to the arsenal of techniques used to elucidate biological roles of metal-Tf binding.

In summary, (i) urea-PAGE is rather unusual for studies of metal speciation but deserves consideration in a range of future studies in similar studies of speciation and changes in protein conformations with changes in experimental conditions. (ii) The results highlight how this technique can provide important insights into understanding the role of transferrin in cellular uptake of biologically or pharmacologically important metal ions.

## 4. Materials and Methods

### 4.1. Reagents and Stock Solutions

Analytical grade (>99% purity) reagents and HPLC grade solvents from Merck were used without further purification, and water was purified by the Milli-Q technique. In order to minimize the influence of residual Fe(III) on metal-Tf binding, buffer solutions were treated with Chelex 100 chelating resin (Bio-Rad, Hercules, CA, USA) for three days, while adjusting pH values with solutions of ultra-pure HCl (0.10 M, Merck, Kenilworth, NJ, USA) or NaOH (0.10 M, Aldrich, Saint Louis, MO, USA). pH values were checked with an Activon 210 ionometer that was equipped with AEP 321 glass/Ag/AgCl electrode and calibrated daily using standard pH solutions (Merck). The purified buffers were filtered through sterile 0.2 μm Minisart RC membrane filters (Sartorius) prior to use. Human apo-Tf (>98% Tf; ≤0.005% Fe; Cat. No. T1147) and holo-Tf (>98% Fe_2_Tf; Cat. No. T4132) from Sigma were used without further purification. 

Stock solutions of Fe(III)-NTA, Cr(III)-NTA, Ga(III)-NTA, In(III)-NTA (NTA = nitrilotriacetic acid), Fe(III)-citrate, Cr(III)-citrate or In(III)-citrate were prepared by mixing aqueous solutions of the corresponding metal nitrates (10 mM metal) with that of NTA (11 mM) or citric acid trisodium salt (22 mM) and then the pH value was adjusted to ~5.0 with aqueous NaOH (1.0 M) [9,10,25,26,49]. The solutions were left to stand for at least a week at 295 K, after which any insoluble metal hydrolysis products that formed were removed using 0.2 μm Minisart RC membrane filters.

Metal concentrations in stock solutions and in purified metal-Tf samples (Section 4.2) were determined by graphite furnace atomic absorption spectroscopy (GFAAS) for Fe, Cr and V and by inductively coupled plasma mass spectrometry (ICP-MS) for Ga and In. An Agilent Technologies Series 200 spectrometer, equipped with Zeeman background correction, was used for GFAAS, and a PerkinElmer Nexion 350X spectrometer was used for ICP-MS. Certified standard metal solutions (1000 ppm) from Choice Analytical were used for calibration, and ICP-MS was internally calibrated using the ^193^Ir peak.

### 4.2. Preparation of Metal-Tf Complexes

Freshly prepared stock solutions of apoTf (100 μM) in the binding buffer (20 mM HEPES, 25 mM NaHCO_3_, 140 mM NaCl, pH 7.4, where HEPES = 4-(2-hydroxyethyl)piperazine-1-ethanesulfonic acid) [3] were used for the preparation of metal-Tf solutions. Stock solutions of monoferric Tf (FeTf) were prepared by mixing 100 μM apoTf with 100 μM Fe(III)-NTA in the binding buffer [9,10,25]. Concentrations of Tf solutions were verified by absorbance measurements at 280 nm with a DeNovix DS-11 FX microvolume spectrophotometer, *ε* (M^−1^ cm^−1^) 8.4 × 10^4^ (apoTf), 9.5 × 10^4^ (FeTf), 10.4 × 10^4^ (Fe_2_Tf) [49]. For the formation of metal-Tf complexes in situ, stock solutions of apoTf or FeTf were diluted with the binding buffer to 30 μM Tf, mixed with a stock metal salt at the required molar ratio and incubated at 310 K for 24 h. Incubation of metal-Tf complexes under endosome-mimicking conditions [3,4,34] was performed at 30 μM Tf in a buffer containing 100 mM MES, 300 mM KCl, 0.10 mM citrate and 1.0 mM ascorbate at pH 5.6 (MES = 2-(*N*-morpholino)ethanesulfonic acid) for 5 min at 295 K. Stock solutions of ascorbic acid (100 mM) were freshly prepared and added to the buffer immediately before the experiment in order to avoid oxidation by air oxygen.

For the experiments with human serum, 60 μL of freshly thawed undiluted serum (Sigma H6914) with or without added metal complexes (50 μM) was incubated for 4 h at 310 K. In order to remove ~90% of albumin from serum, the samples were diluted with 240 μL of low-salt buffer (20 mM HEPES, pH 7.4) and passed through Affi-Gel Blue columns (BioRad Cat. No. 7326708) that were pre-saturated with the same buffer. The columns were then washed with 400 μL of the same buffer, and a 10 μL aliquot of the pooled sample was used for loading into the gel (Section 4.3).

Purified samples of Ga_2_Tf, GaFeTf, In_2_Tf and InFeTf were prepared by reactions of 100 μL of 100 μM apoTf or FeTf solutions with 10-fold (apoTf) or 5-fold (FeTf) molar excess of Ga(III)-NTA or In(III)-NTA solutions in the binding buffer for 24 h at 295 K, followed by the removal of unbounded metal ions with Micro BioSpin gel filtration columns (Bio-Rad Cat. No. 7326321; molecular mass cutoff, 30 kDa) that were pre-saturated with the same buffer [9,10]. Due to the air-sensitivity of V(III) and V(IV) complexes [19,20], samples of V^III^_2_Tf, V^III^FeTf, V^IV^_2_Tf and V^IV^FeTf were prepared in a glovebag filled with high purity Ar. Small aliquots (~0.1 mg) of solid VCl_3_ or VOSO_4_·5H_2_O were dissolved in 0.50 mL of 100 μM apoTf or FeTf solutions in 100 mM HEPES, 25 mM NaHCO_3_ buffer (pH 7.4) and kept under an Ar atmosphere for 24 h at 295 K. The resulting mixtures were centrifuged (5 min at 16,000× *g*) in order to remove any precipitate and passed through gel filtration columns (Bio-Rad Cat No. 7326321, pre-saturated with the binding buffer) under ambient atmosphere. Purified solutions of V(III)-Tf complexes were stable for at least a week under ambient atmosphere at 277 K (determined by urea-PAGE gel electrophoresis, see Figure 5 and Figure S1).

The purity of the metal-Tf complexes after gel filtration was verified by UV-vis spectroscopy (Figure S3 in Supplementary Material) and by the measurements of metal/protein ratio. For the latter, an aliquot of the purified solution (10 μL) was digested with 0.20 mL of 69% HNO_3_ (Merck ultra-pure grade), and metal content was determined by GFAAS or ICP-MS. Another aliquot of the solution was used for protein determination with Bradford reagent (Sigma Cat. No. B6916) with apoTf as the calibration standard. The determined metal/protein molar ratios were within 20% of the expected values.

### 4.3. Urea-PAGE Gel Electrophoresis

Standard TBE-Urea gel conditions (TBE is Tris-borate-EDTA buffer, where Tris is tris(hydroxymethyl)aminomethane, and EDTA = *N*,*N*,*N*′*N*′-ethanediaminetetraacetic acid) [50] were used. Solutions of metal-Tf complexes were diluted 6-fold with either the binding buffer (pH 7.4) or the endosomal buffer (pH 5.6) immediately prior to loading into the gel. The dilute samples (10 μL) were mixed 1:1 with TBE-Urea sample buffers (Thermo Fisher Scientific, Cat.No. LC6876, Waltham, MA, USA) before loading onto the gel (6% TBE-Urea, 10 wells, EC6865). The running buffer was 89 mM Tris base, 89 mM boric acid, 2.0 mM EDTA free acid, pH 8.3 [50]. Electrophoresis was performed for 3 h at 180 V, the bands were visualized with EZBlue gel staining reagent (Sigma G1041) for 5 min at 295 K and then gel was destained with water for 15 min. The gels were photographed using the BioRad ChemiDoc MP imaging system, and the images were processed using BioRad ImageLab 5.2 software. For each condition, qualitative agreement was obtained in at least two independent experiments using different preparations of metal-Tf complexes, as illustrated in Figure 5 and Figure S1.

## Data Availability

Primary research data for this project are stored in the Electronic Laboratory Notebooks of Aviva Levina and Boer Wang administered by the University of Sydney.

## Supplementary Materials

The following are available online at https://www.mdpi.com/article/10.3390/gels8010019/s1, Figure S1: Additional gel electrophoresis data for V^III^_2_Tf and V^III^FeTf complexes. Figure S2: Processing of gel electrophoresis results for the reaction of human serum with metal complexes; Figure S3: UV-vis spectra of purified metal-Tf solutions.
