# Supplementary material for: Urea Gel Electrophoresis in Studies of Conformational Changes of Transferrin on Binding and Transport of Non-Ferric Metal Ions
Source: Gels. 2021 Dec 27;8(1):19. doi: 10.3390/gels8010019 (PMC8774473; doi:10.3390/gels8010019)
Supplement: Supplementary file 1 [file gels-08-00019-s001.zip › gels-1510518-supplementary.pdf]

Urea Gel Electrophoresis in Studies of Conformational Changes of Transferrin on Binding and Transport of Non-Ferric Metal Ions

Aviva Levina*, Boer Wang and Peter A. Lay*

Supplementary Material

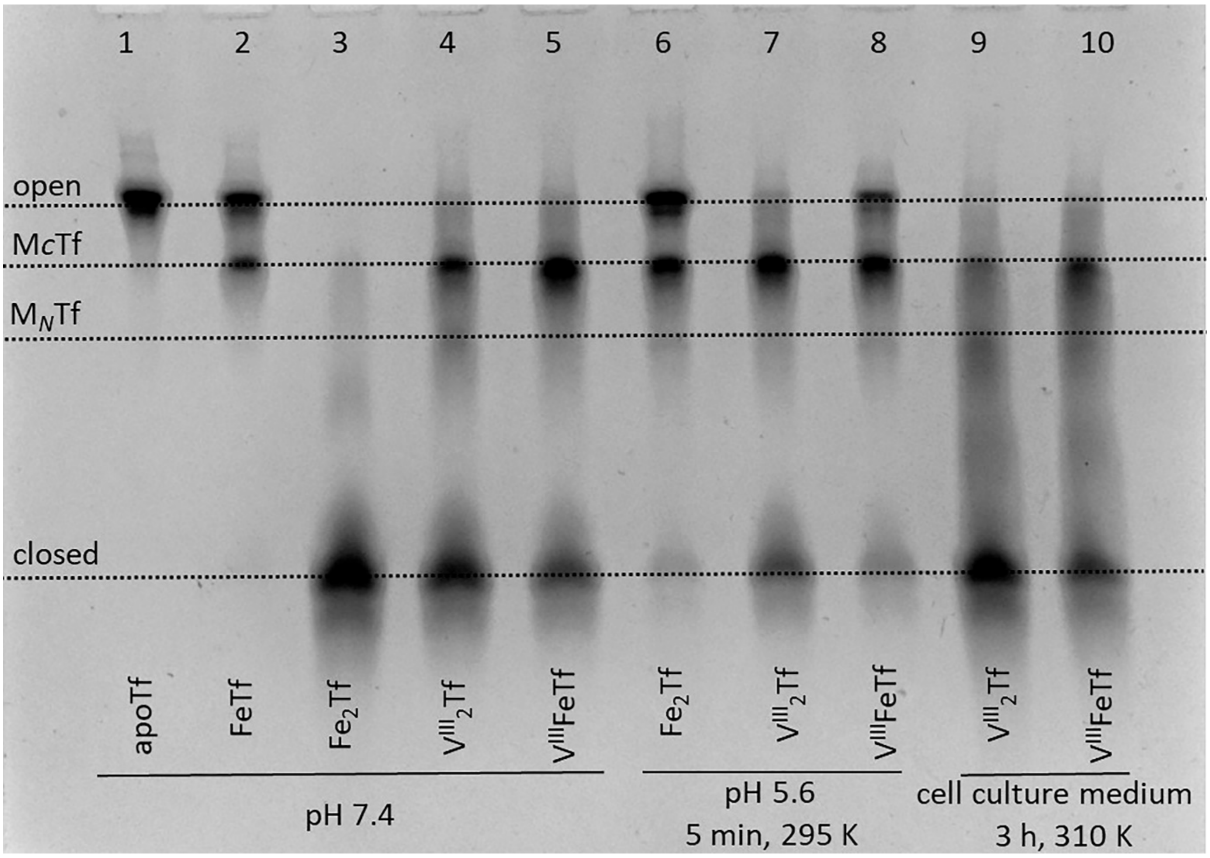

Figure S1. Urea-PAGE of the purified samples of $V^{III}{}_2Tf$ and $V^{III}FeTf$ and control samples under the conditions mimicking the extracellular (lanes 1-5; 20 mM HEPES, 25 mM $NaHCO_3$, 140 mM NaCl, pH 7.4, 295 K) and endosomal (lanes 6-8; 100 mM MES, 300 mM KCl, 0.10 mM citrate, 1.0 mM ascorbate, pH 5.6, 5 min at 295 K) stages of the Tf cycle [3], as well as cell culture conditions (lanes 9-10; Advanced DMEM with 2% fetal calf serum, 3 h at 310 K at 5% CO_2) [10]. Initial Tf concentration in the reaction mixtures was 30 μM, and the samples were diluted 6-fold with the corresponding buffers before loading into the gel. Four main Tf conformations (Figure 1 in the main text) are marked (M_CTf and M_NTf contain one metal ion bound to *C*- or *N*-lobes, respectively). Samples of $V^{III}{}_2Tf$ and $V^{III}FeTf$ were prepared as described in Materials and Methods (main text), except that the reaction mixture were kept under Ar atmosphere at 295 K for 1 h, rather than 24 h. Lanes 4 and 5 demonstrate the formation of a mixture of fully closed and partially open Tf conformations under these conditions (compare with Figure 5 in the main text). Lanes 6-8 demonstrate the lower efficiency of dissociation of V(III), compared with Fe(III), under the endosomal condition (same as in Figure 5). Lanes 9 and 10 demonstrate the partial stability of the closed V(III)-Tf conformation under cell culture conditions. .

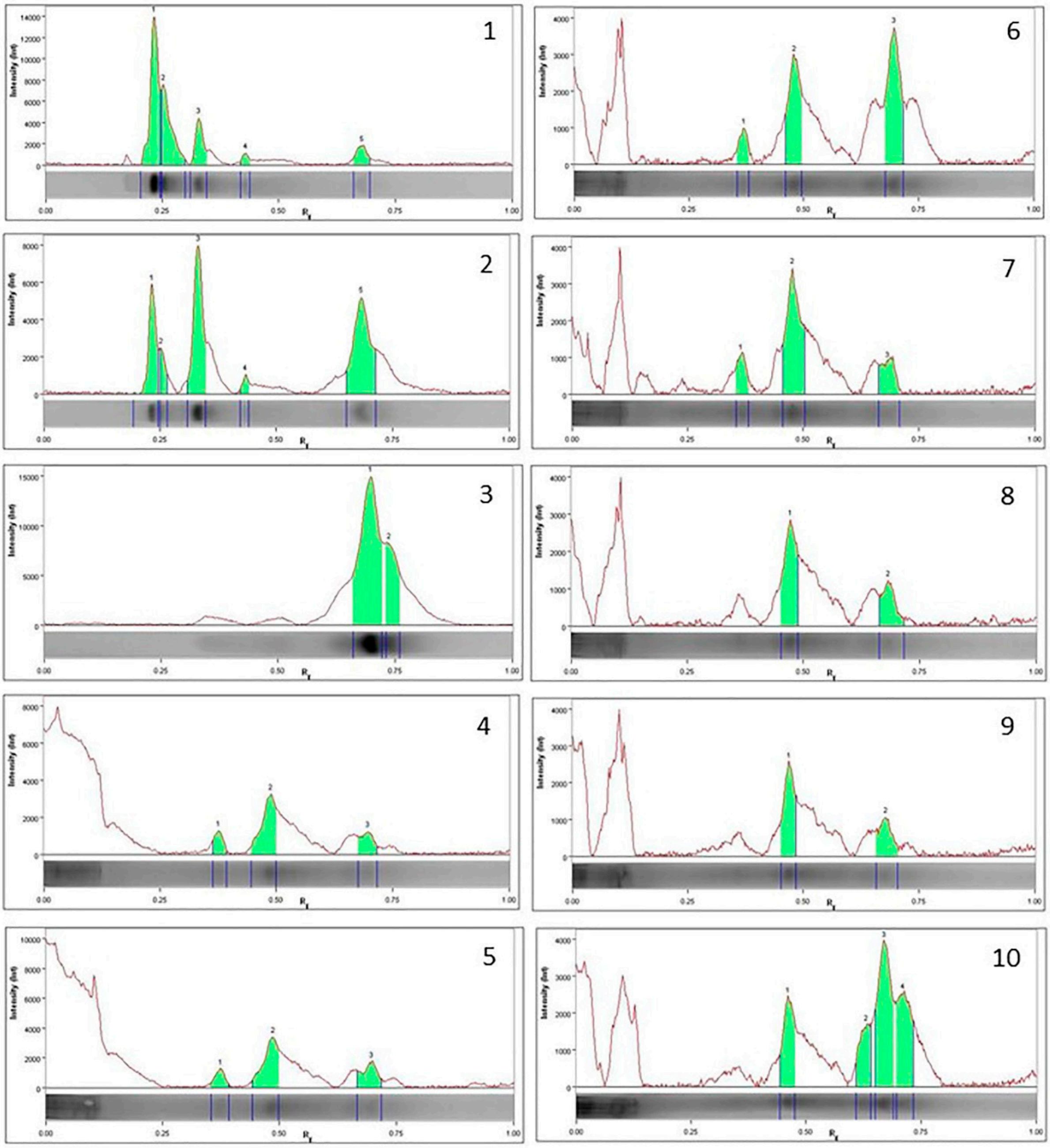

Figure S2. Integrated band intensities, calculated with BioRad ImageLab 5.2 software, for the gel shown in Figure 6, main text. Lane numbers are shown in the top right corner of each figure.

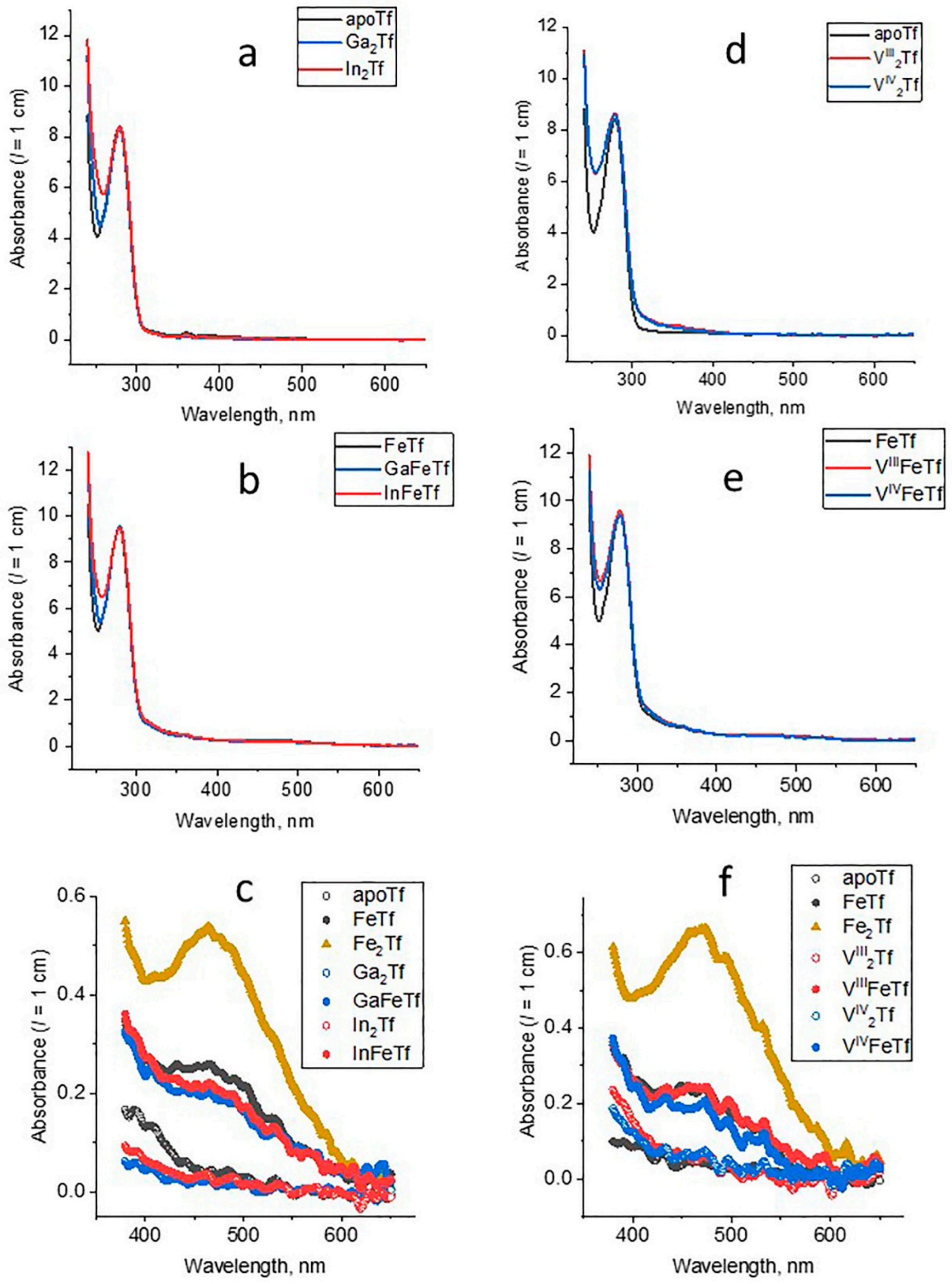

Figure S3. UV-vis spectra of metal-Tf samples after purification by gel filtration chromatography: (**a**-**c**) Ga(III)-Tf and In(III)-Tf; (**d**-**f**) V(III)-Tf and V(IV)-Tf (see Section 4.2 in the main text). Spectra were collected with DeNovix DS-11 FX spectrophotometer (sample size, 2 µL). Absorbance was corrected for 1 cm pathlength and normalized for 100 µM Tf, using the protein concentrations determined with Bradford reagent. Solutions of apoTf, FeTf and Fe_2Tf (100 µM each) in the binding buffer (20 mM HEPES, 25 mM $NaHCO_3$, 140 mM NaCl, pH 7.4) were used as controls. Expanded spectral regions at 380-650 nm, corresponding to the Fe(III)-Tf absorbance bands [25], are shown in (**c**) and (**f**).
